# Evaluation of automatic contour propagation in T2‐weighted 4DMRI for normal‐tissue motion assessment using internal organ‐at‐risk volume (IRV)

**DOI:** 10.1002/acm2.12431

**Published:** 2018-08-15

**Authors:** Jingjing Zhang, Svetlana Markova, Alejandro Garcia, Kirk Huang, Xingyu Nie, Wookjin Choi, Wei Lu, Abraham Wu, Andreas Rimner, Guang Li

**Affiliations:** ^1^ Department of Radiation Oncology Zhongshan Hospital of Sun Yat‐Sen University Zhongshan China; ^2^ Department of Medical Physics Memorial Sloan Kettering Cancer Center New York New York; ^3^ Department of Radiation Oncology Memorial Sloan Kettering Cancer Center New York New York

**Keywords:** deformable image registration and automatic contour propagation, four‐dimensional magnetic resonance imaging, normal tissue and organ contouring, respiratory‐induced organ motion, treatment planning

## Abstract

**Purpose:**

The purpose of this study was to evaluate the quality of automatically propagated contours of organs at risk (OARs) based on respiratory‐correlated navigator‐triggered four‐dimensional magnetic resonance imaging (RC‐4DMRI) for calculation of internal organ‐at‐risk volume (IRV) to account for intra‐fractional OAR motion.

**Methods and Materials:**

T2‐weighted RC‐4DMRI images were of 10 volunteers acquired and reconstructed using an internal navigator‐echo surrogate and concurrent external bellows under an IRB‐approved protocol. Four major OARs (lungs, heart, liver, and stomach) were delineated in the 10‐phase 4DMRI. Two manual‐contour sets were delineated by two clinical personnel and two automatic‐contour sets were propagated using free‐form deformable image registration. The OAR volume variation within the 10‐phase cycle was assessed and the IRV was calculated as the union of all OAR contours. The OAR contour similarity between the navigator‐triggered and bellows‐rebinned 4DMRI was compared. A total of 2400 contours were compared to the most probable ground truth with a 95% confidence level (S95) in similarity, sensitivity, and specificity using the simultaneous truth and performance level estimation (STAPLE) algorithm.

**Results:**

Visual inspection of automatically propagated contours finds that approximately 5–10% require manual correction. The similarity, sensitivity, and specificity between manual and automatic contours are indistinguishable (*P* > 0.05). The Jaccard similarity indexes are 0.92 ± 0.02 (lungs), 0.89 ± 0.03 (heart), 0.92 ± 0.02 (liver), and 0.83 ± 0.04 (stomach). Volume variations within the breathing cycle are small for the heart (2.6 ± 1.5%), liver (1.2 ± 0.6%), and stomach (2.6 ± 0.8%), whereas the IRV is much larger than the OAR volume by: 20.3 ± 8.6% (heart), 24.0 ± 8.6% (liver), and 47.6 ± 20.2% (stomach). The Jaccard index is higher in navigator‐triggered than bellows‐rebinned 4DMRI by 4% (*P *< 0.05), due to the higher image quality of navigator‐based 4DMRI.

**Conclusion:**

Automatic and manual OAR contours from Navigator‐triggered 4DMRI are not statistically distinguishable. The navigator‐triggered 4DMRI image provides higher contour quality than bellows‐rebinned 4DMRI. The IRVs are 20–50% larger than OAR volumes and should be considered in dose estimation.

## 1 INTRODUCTION

Respiration‐induced tumor motion is a major source of uncertainties in radiotherapy of thoracic and abdominal cancer.^1^, ^2^, ^3^ The motion of the nearby organ at risk (OAR) relative to the tumor plays an important role in radiation toxicity, which may become a limiting factor to prescribing an effective ablative dose for stereotactic body radiotherapy (SBRT).^4^, ^5^ Clinically, the patient respiratory motion is assessed with respiratory‐correlated four‐dimensional computed tomography (4DCT) using an external respiratory surrogate. The internal tumor volume (ITV), the union of clinical tumor volumes in all respiratory phases, is the recommended target by the International Commission on Radiation Units and Measurements (ICRU 50 and 62) as the treatment target to account for respiratory‐induced tumor motion. Alternatively, the ITV has been also obtained via generating maximum intensity projection (MIP) image.^6^, ^7^, ^8^ The full prescription dose is planned to cover the planning tumor volume (ITV+margin), which may include nearby OAR causing normal tissue toxicity.^4^ A planning organ‐at‐risk volume (PRV)^9^, ^10^, ^11^ is used to account for inter‐fractional variation in OAR position, but not intra‐fractional motion, therefore, the OAR motion has not been properly accounted for, leading uncertainties in OAR dose estimation, treatment toxicity,^12^, ^13^ and the dose‐toxicity relationship.^4^, ^14^ On the contrary, incorporating OAR motion in treatment planning may help to provide an improved OAR dose estimation and therefore optimized dose prescription for an SBRT treatment. More accurate clinical data may lead to better understanding of SBRT dose‐limiting toxicity^15^, ^16^, ^17^, ^18^ and normal tissue complication probabilities (NTCP),^13^ to improve the therapeutic ratio.

Respiratory‐correlated 4D Magnetic resonance imaging (4DMRI) offers higher soft‐tissue contrast^19^, ^20^, ^21^, ^22^, ^23^ and fewer binning artifacts using an internal navigator echo rather than an external surrogate,^23^ which is used by 4DCT. In addition, time‐resolved 4DMRI over multi‐breathing cycles have also been reported, producing more than 10‐phase clinical motion data.^24^, ^25^, ^26^ Because four‐dimensional MRI is an emerging 4D imaging modality with great potential benefits in radiotherapy applications, it is of paramount importance to perform the preclinical evaluation, especially more imaging data, prior to clinical use in radiotherapy planning. Automatic contour propagation using deformable image registration (DIR) was reported for 4DCT‐based respiratory motion assessment,^27^, ^28^, ^29^ CT‐based longitudinal adaptive evaluation,^30^, ^31^, ^32^ and cone‐beam CT for setup with liver matching.^33^ Other automatic image segmentation approaches were reported,^34^, ^35^ including model‐based method. Recently, automatic OAR contouring on 2D cine or 3D MR images were reported to facilitate MR‐based planning or MR‐guided radiotherapy.^36^, ^37^, ^38^, ^39^ With both high soft‐tissue contrast and low binning artifacts, T2W navigator‐triggered 4DMRI provides more anatomic landmarks than 4DCT, facilitating DIR for automatic contour propagation for OAR motion assessment for treatment planning and delivery.

To evaluate the accuracy of an automatic segmentation method in human images, physician's manual contours are used as the clinical ground truth. However, the intra‐ and inter‐observer variation is common, leading to multiple ground truths. To minimize the uncertainty in the scientific ground truth, the simultaneous truth and performance level estimation **(**STAPLE) algorithm was developed^40^ and applied to evaluate tumor delineation in radiotherapy planning of lung, liver, and pancreatic cancer using CT or 4DCT.^41^, ^42^, ^43^, ^44^ Based on the statistics of a group of clinical ground truths from multiple physicians, the most probable ground truth can be computed and used as the scientific ground truth.

In this study, automatic OAR contour propagation of T2W 4DMRI was evaluated for OAR delineation and generation of internal organ‐at‐risk volume (IRV) to account for intra‐fractional OAR motion, as the PRV only accounts for inter‐fractional OAR motion. Ten volunteers were scanned for navigator‐triggered T2W 4DMRI (10 bins) under an IRB‐approved protocol and four major organs (lungs, heart, liver, and stomach) were segmented manually and automatically by a radiation oncologist and a trained medical student. We hypothesize that the DIR‐propagated contours from the 4DMRI images have similar quality to the manual contours. The quality of automatically propagated contours was evaluated using the most probable ground truth with a 95% confidence level (S95) using the STAPLE algorithm.^40^ The OAR contour quality was also evaluated using navigator‐triggered and bellows‐rebinned 4DMRI. Finally, the volume increase from OAR to IRV was quantified using IRV/V^OAR^ ratio.

## 2 METHODS AND MATERIALS

### 2.1 Respiratory‐correlated 4DMRI image acquisition

T2W respiratory‐correlated (RC) 4DMRI images of 10 healthy volunteers were acquired in a 3T MR scanner (Ingenia, Philips Healthcare) in coronal directions using an internal navigator as the respiratory surrogate under an IRB‐approved protocol. The bellows waveform was collected simultaneously for retrospective reconstruction of bellows‐rebinned 4DMRI. The navigator echo window (3 × 3 × 6 cm^3^) was placed on the right diaphragm dome and amplitude‐binning was used for 4D image reconstruction.^23^ Ten‐respiratory bins were used in all 4DMRI reconstructions. The pulse sequence used in 4DMRI scanning included turbo spin echo with, TE/TR of 80/6000 ms, flip angle of 90°, and pixel bandwidth of 470 Hz. To avoid signal saturation, 4–8 packs of acquisition (segmented acquisition bands) were defined to ensure two consecutive 2D slice images were acquired from different packs. The 4D scanning program used the first 10s breathing waveforms as a training dataset for amplitude‐based binning for the rest of the scan until the bin‐slice array (table) was filled. The images have a pixel size of 2 × 2 mm^2^ and slice spacing of 5 mm. The 4DMRI acquisition lasted 6–15 minutes with a large field of view covering the lungs, stomach, and liver. The navigator‐triggered 4DMRI images were rebinned using the concurrent bellows waveforms (bellows‐rebinned)^23^ for contour delineation and comparison.

### 2.2 Manual and automatic delineation of the normal structures

Based on the T2W navigator‐triggered 4DMRI images, a radiation oncologist and a medical student contoured five OARs, including the right and left lungs, heart, liver, and stomach, in each respiratory phase using an in‐house treatment planning system (Metropolis). A written guideline of segmentation was provided, including the window/level settings (0–1200 for T2w 4DMRI) and the anatomic landmark to define the superior end of the heart (defined as when the two ascending arteries split in axial view). The intra‐observer variability was examined based on volume variation among ten breathing phases on volume‐preserved organs, such as the heart, liver, and stomach. The OAR contours based on navigator‐triggered 4DMRI was compared with bellows‐rebinned 4DMRI and the difference was assessed. Rigid alignment between the two sets of 4DMRI images was performed prior to the contour delineation and comparison.

A fast free‐form multi‐resolution DIR method^45^, ^46^ was employed to propagate the contour from the full‐exhalation phase to the other respiratory phases. An intensity‐based metric was used as the registration criterion to minimize an energy function that accounts for voxel intensity similarity and smoothness between two images:

(1) $$E\left(\right.u)=\int \left(\right.I_{B}\left(\right.\vec{x}+\vec{u}))-I_{A}\left(\right.\vec{x}){)}^{2}d\vec{x}+\lambda \sum_{i=1}^{3}\int {\left|\nabla u_{i}\right|}^{2}d\vec{x}$$

The first term describes the voxel intensity (*I*_*A*_ and *I*_*B*_) between images A and B at point $\vec{x}$, while the second term is for smoothing that regulates the gradient changes in the vector displacement field $\vec{u}$. The *λ *= 0.1 parameter sets the weighting factor between the two terms. The displacement vector field is found by solving the Euler‐Lagrange equation through an iterative approach:

(2) $$\lambda \nabla^{2}u^{2}+\left(I_{B}\left(\vec{x}+\vec{u}\right)-I_{B}\left(x\right)\right)\frac{\partial I_{B}\left(\vec{x}+\vec{u}\right)}{\partial \vec{u}}=0$$

Using the same window/level settings with optimal OAR visualization for both moving and fixed MRI images. The region of interest was drawn to cover the anatomy of interest, excluding surrounding most air voxels outside the body, and the displacement vector field generated from DIR was applied for contour propagation. Two sets of DIR‐propagated and two sets of manual contours in all respiratory states were generated, compared, and analyzed using the method described below. The accuracy of the free‐form DIR was previously found to be ~3.5 mm using 4DCT of a deformable phantom.^46^ It is expected that the DIR accuracy for 4DMRI with better soft‐tissue contrast and low binning artifacts should be no worse than 4DCT.

### 2.3 Assessment of DIR‐propagated OAR contours using the STAPLE algorithm

A statistical analysis of multi‐sets of contours was conducted using the STAPLE algorithm^40^ to provide similarity (Jaccard index), sensitivity (true positive), and specificity (true negative)^44^, ^47^ (or SSS), which were expressed as:

(3) $$\text{Jaccard}=\frac{D\cap G}{D\cup G},\text{Sensitivity}=\frac{D\cap G}{G},\text{Specificity}=\frac{\overset{¯}{D}\cup \overset{¯}{G}}{\overset{¯}{G}}$$

where *D* and *G* are the volumes enclosed inside individual and group consensus contours, while $\overset{¯}{D}\;\;and\;\;\overset{¯}{G}$ are the space outside of the volumes D and G, respectively. The STAPLE calculated the most probable ground truth with a 95% confidence level (S95) using a maximum likelihood algorithm based on input contours, which are assumed to be close to the ground truth. Four manual and automatic contour sets were used and evaluated against the S95 contour. The Student's t‐test was performed after the STAPLE analysis for the *P*‐value of the SSS results between the manual and automatic contours and between the contours from navigator‐triggered and bellows‐rebinned 4DMRI images.

The STAPLE algorithm was implemented in python script language. Based on the contour inputs (two manual contours or two DIR‐propagated contours checked by visual and corrected as needed), the program first generated a probability map, then calculated the most probable ground truth (S95) at 95% confidence level as the reference ground truth to evaluate the similarity, sensitivity, and specificity of the input individual contours using Eq.(3).

### 2.4 OAR volume variation and internal organ‐at‐risk volume

The volume‐conserving organs, such as the heart, liver, and stomach, do not change their volume with the respiratory motion, although they may deform, because non‐lung tissues are not compressible under respiratory pressure difference (3–6 mmHg).^48^ Therefore, the delineated OAR volumes are expected to be constant. The variation in delineated OAR volumes throughout the breathing cycle was compared and analyzed, based on the same 4DMRI image sets or different 4DMRI image sets (navigator‐triggered vs. bellows‐rebinned).

The IRV, defined as the union of OAR contours in all phases, was created using Boolean (OR) operation to evaluate OAR volume increase due to the motion. The IRVs from both manual and automatic contours were calculated for heart, liver, and stomach, and compared to the mean OAR volume using$\;\;\%V=\left(\frac{\mathrm{IRV}}{{\overset{¯}{V}}^{\mathrm{OAR}}}-1.0\right)\times 100$. The mean OAR volume ( ${\overset{¯}{V}}^{\mathrm{OAR}}$) was calculated from those in all respiratory phases.

## 3 RESULTS

### 3.1 Intra‐ and inter‐observer variability in OAR contours using 4DMRI

**Figure 1:**
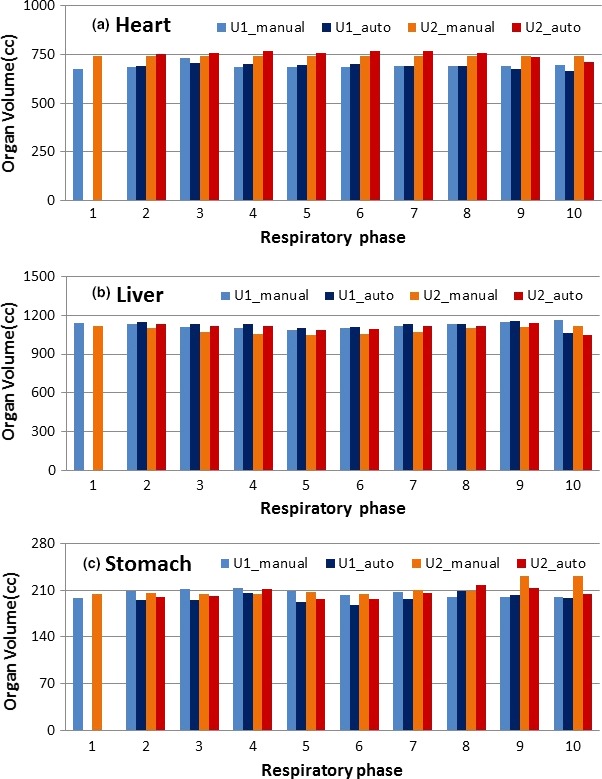
A typical example (volunteer 5) of manual vs. automatic contours and intra‐ and inter‐observer variability in three volume‐conserving organs based on T2W RC‐4DMRI. The auto‐contour variation is smaller than inter‐observer variation (U1 = user1 and U2 = user2).

**Figure 2:**
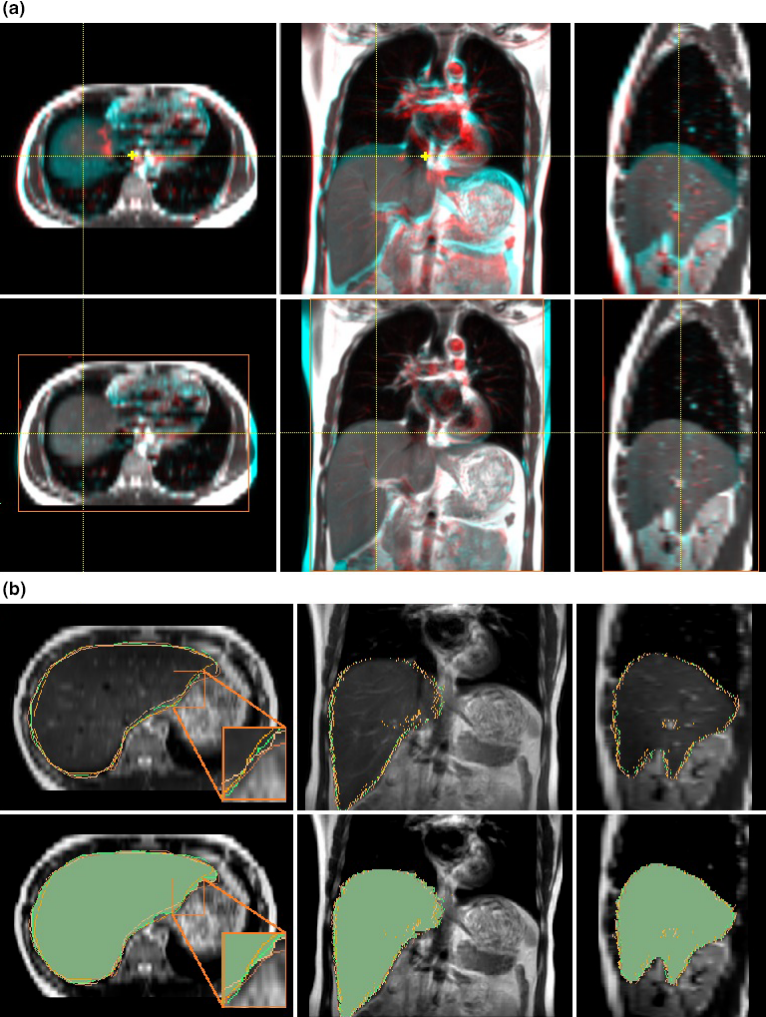
(a) Deformable image registration between full exhalation (blue) and full inhalation (red) of T2W navigator‐triggered 4DMRI (volunteer #7). Soft‐tissue alignment before and after DIR within a region of interest (orange box) makes aligned voxel (white), except for the flowing blood voxels inside the major vessels around the heart. (b) Comparison of four sets of manual and auto contours (user1: orange/green, user2: pink/brown) in the upper panel and with the most probable ground truth (S95, green area), generated by the STAPLE, in the lower panel.

The intra‐observer variability was assessed using volume‐conserved organs, including the heart, liver, and stomach. Fig. 1 demonstrates a volunteer example of the intra‐observer variability in three manually contoured organ volumes. The small intra‐observer variability (±3%) is found due to the high soft‐tissue contrast of T2W 4DMRI images. The results for all 10 volunteers are tabulated in Table 1. The changes in the center of mass of the liver and stomach are smaller than the diaphragm motions on the same side. For the liver, the manual contours are close to the ground truth, as shown in Fig. 2.

**Table 1:** Evaluation of intra‐observer variability in manual contours of three volume‐conserving organs (heart, liver, and stomach) based on relative volume change within a breathing cycle

Subject^a^	Diaphragm motion (cm)^b^		OAR motion, COM (cm)^c^		Intra‐observer variability (volume variation, %V)^d^			Inter‐observer variability (similarity variation, %S)^e^		
	Right	Left	Liver	Stomach	Heart	Liver	Stomach	Lungs	Heart	Liver
1	1.2	1.5	–	–	1.9	–	–	7.4	−8.2	–
2	1.4	1.7	0.7	0.5	2.0	1.5	3.0	5.1	−4.6	7.9
3	1.5	1.0	1.2	0.8	1.1	0.7	2.6	8.7	9.2	7.0
4	1.0	2.0	1.0	1.4	3.1	0.9	4.0	6.6	−6.4	7.2
5	1.6	1.1	0.7	0.7	2.6	2.4	3.3	1.1	3.3	−0.3
6	1.6	1.4	1.0	1.0	2.1	1.1	2.1	4.1	3.1	3.0
7	1.2	2.3	1.4	2.0	2.0	0.8	2.7	7.0	−7.0	5.9
8	2.6	2.4	2.3	2.4	2.7	1.7	2.3	5.6	9.2	13.3
9	1.2	1.5	0.9	1.2	1.5	0.8	1.2	–	8.6	8.1
10	2.5	1.8	1.6	1.5	6.2	1.1	2.0	1.3	−9.6	1.7
Mean	1.6	1.7	1.2	1.3	2.6	1.2	2.6	5.2	−2.0	6.0
SD	0.6	0.5	0.5	0.6	1.5	0.6	0.8	2.6	7.1	4.1

a Volunteers 1 and 9 have insufficient inferior and superior field of view, respectively.

b The diaphragm motion range (or maximum displacement).

c Organ at risk (OAR) motion refers to COM (center of mass) trajectory in the sup‐inf direction.

d These OARs are considered volume‐conserving organs (%V = SD/mean × 100%).

e Relative difference of averaged similarity (Jaccard index) [%S = 2∙(S^U1^‐S^U2^)/(S^U1^+S^U2^) × 100%]. The mean similarity values are >0.9 (See Table 2). U1 = User 1 and U2 = User 2.

### 3.2 Comparison of manual and automatic OAR contours

The automatically propagated contours among 4DMRI are based on DIR that should have no worse than the uncertainty of ~3.5 mm, which was validated in 4DCT, due to the high MR soft‐tissue contrast and minimal binning artifacts of the navigator‐triggered 4DMRI, as shown in Fig. 2A. As a result, automatic contours are very similar to the manual contours from the same observer (Fig. 2B). It is worthwhile to mention that there are ~5–10% outliers with obvious flaws in the propagated contours, often occurring at superior‐inferior OAR edges, and visual checking and manual correction are necessary. Fig. 2B shows the S95 contour in a liver case, generated from two manual and two auto high‐quality contours using the STAPLE.

Table 2 tabulates the similarity, sensitivity, and specificity between manual and automatic contours. The similarity between manual and automatic contours from the same observer is generally greater than that of inter‐observers, as shown in Tables 1 and 2, suggesting high‐quality automatic OAR contours. The sensitivity and specificity of the manual and automatic contours are similarly high in comparison with the S95 contour.

**Table 2:** Comparison of manual and automatic contours with the most probable ground truth (S95) contour based on their similarity (Jaccard Index), sensitivity, and specificity averaged from ten respiratory phases and two observers on four OARs. The manual and automatic contours are statistically indistinguishable

STAPLE a assessment	Subject^b^	Lungs (L+R)		Heart		Liver		Stomach	
		Manual	Auto	Manual	Auto	Manual	Auto	Manual	Auto
Similarity	1	0.91	0.91	0.89	0.88	–	–	–	–
(Jaccard)	2	0.92	0.90	0.91	0.92	0.89	0.90	0.84	0.83
Index)	3	0.91	0.91	0.93	0.86	0.91	0.91	0.85	0.81
	4	0.94	0.91	0.88	0.87	0.92	0.91	0.85	0.83
	5	0.92	0.91	0.89	0.96	0.93	0.95	0.79	0.83
	6	0.93	0.94	0.92	0.90	0.92	0.93	0.76	0.78
	7	0.93	0.93	0.89	0.89	0.94	0.95	0.90	0.91
	8	0.93	0.94	0.89	0.91	0.87	0.89	0.78	0.75
	9	–	–	0.87	0.89	0.91	0.91	0.83	0.87
	10	0.92	0.92	0.87	0.84	0.91	0.91	0.83	0.82
	Average	0.92	0.92	0.89	0.89	0.91	0.92	0.82	0.83
	SD	0.02	0.01	0.02	0.04	0.02	0.02	0.04	0.04
	*P*‐value	0.35		0.79		0.08		1.00	
Sensitivity	1	0.93	0.93	0.93	0.90	–	–	–	–
	2	0.94	0.94	0.95	0.95	0.93	0.93	0.87	0.85
	3	0.93	0.94	0.96	0.96	0.93	0.93	0.91	0.87
	4	0.95	0.93	0.91	0.91	0.94	0.92	0.89	0.87
	5	0.94	0.93	0.94	0.97	0.94	0.96	0.83	0.85
	6	0.94	0.95	0.96	0.91	0.94	0.94	0.81	0.80
	7	0.95	0.95	0.92	0.91	0.95	0.96	0.94	0.93
	8	0.94	0.95	0.92	0.93	0.90	0.92	0.84	0.80
	9	–	–	0.92	0.92	0.93	0.93	0.88	0.90
	10	0.94	0.95	0.91	0.88	0.93	0.94	0.87	0.86
	Average	0.94	0.94	0.93	0.92	0.93	0.94	0.87	0.86
	SD	0.02	0.01	0.03	0.04	0.01	0.01	0.04	0.04
	*P*‐value	0.76		0.44		0.31		0.13	
Specificity	1	0.98	0.97	0.95	0.97	–	–	–	–
	2	0.94	0.94	0.98	0.97	0.93	0.93	0.96	0.97
	3	0.93	0.94	0.96	0.90	0.93	0.93	0.93	0.93
	4	0.98	0.98	0.97	0.96	0.98	0.98	0.95	0.94
	5	0.97	0.98	0.95	0.98	0.98	0.99	0.94	0.97
	6	0.99	0.98	0.96	0.99	0.98	0.98	0.93	0.97
	7	0.98	0.98	0.96	0.97	0.99	0.99	0.96	0.98
	8	0.98	0.97	0.97	0.98	0.97	0.96	0.91	0.93
	9	–	–	0.95	0.96	0.97	0.98	0.93	0.97
	10	0.98	0.97	0.95	0.96	0.97	0.97	0.94	0.95
	Average	0.98	0.97	0.96	0.96	0.97	0.97	0.94	0.96
	SD	0.03	0.02	0.03	0.04	0.02	0.02	0.02	0.02
	*P*‐value	0.45		0.64		0.59		0.01^c^	

a STAPLE refers to the simultaneous truth and performance level estimation algorithm.

b Volunteers 1 and 9 have an incomplete field of view in the inferior and superior, respectively.

c The *P* < 0.05 indicates that the automatic contour is slightly better than the manual contour.

### 3.3 Organ motion in 4DMRI and internal organ‐at‐risk volume

**Figure 3:**
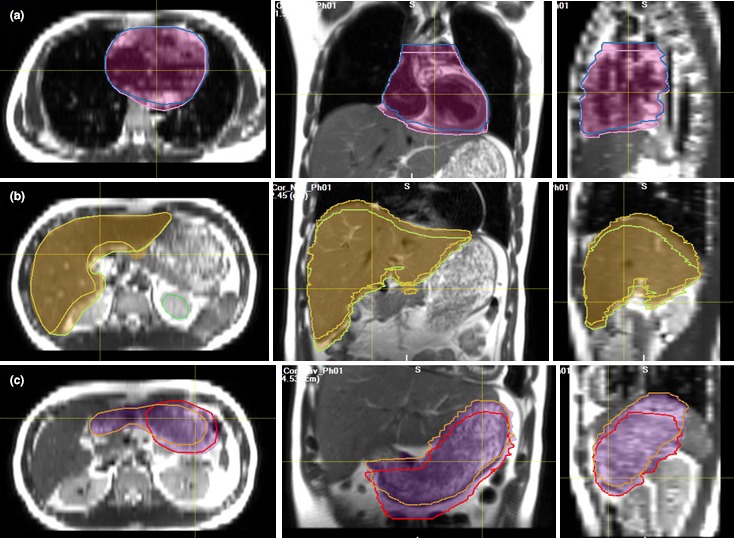
Visual illustration of the internal organ at risk volume (IRV, color shaded) and individual organ at risk (OAR) contour volumes for volunteer #7 in full exhalation and inhalation phases: (a) heart, (b) liver, and (c) stomach. On average, the volume increase from the OAR volume to IRV is 20–50%, depending on the OAR motion, volume, and contour accuracy.

The OAR motion can be represented by their center of mass trajectory within the breathing cycle. The superior‐to‐inferior motions of the liver and stomach are listed in Table 1, together with those of the left and right diaphragm domes. When considering OAR motion, the IRV is shown in Figure 3. For all 10 subjects, the IRV volume increases from the OAR volume by 20.3 ± 8.6%, 24.0 ± 8.6%, and 47.6 ± 20.2% for the heart, liver, and stomach, respectively. At the superior and inferior border of an OAR, the organ tissue voxels only periodically occupy the space, and therefore the IRV in that region decreases its duration from 100% to 0%, appearing blurred its appearance in the averaged MR images from 4DMRI.

### 3.4 Contour differences in 4DMRI reconstructed using internal and external surrogates

**Figure 4:**
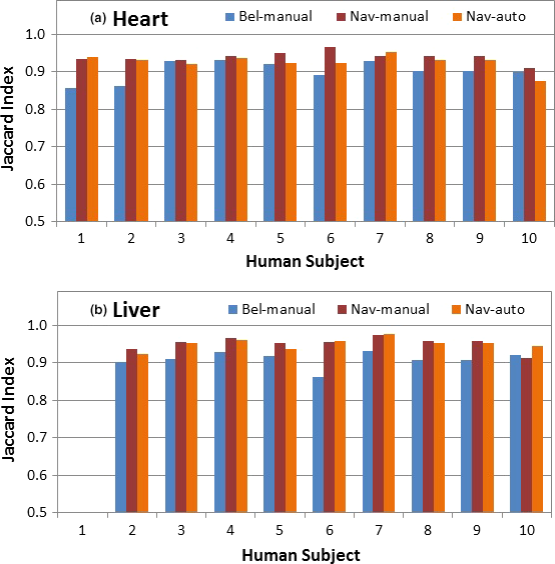
Variation in heart and liver contour similarity based on the navigator‐triggered and bellows‐rebinned 4DMRI images. The similarity difference between manual and automatic contours (heart: *P* = 0.003 and 0.042 and liver: *P* = 0.001 and 0.001, respectively) in Navigator‐based and Bellows‐based 4DMRI is statistically significant.

The manual contours based on navigator‐ and bellows‐binned 4DMRI are compared in the heart and liver for clinically relevant differences. Figure 4 depicts that both the manual and automatic contours based on bellows‐rebinned 4DMRI are consistently inferior to the navigator‐triggered 4DMRI, reflecting image quality difference. The *P*‐values (*P* < 0.05) are shown in Fig. 4, indicating statistically significant difference: both manual and automatic contours have higher similarity to S95 contours in Navigator‐binned than those in Bellows‐binned 4DMRI.

## 4 DISCUSSION

### 4.1 The high soft‐tissue contrast in T2W navigator‐triggered 4DMRI

Because of high soft‐tissue contrast and low artifacts in T2W 4DMRI reconstructed based on an internal navigator,^23^ these features facilitate both manual and automatic segmentation. The quality of automatic contours is acceptable, demonstrated by their high similarity, sensitivity, and specificity to the S95 contours, generated by the STAPLE software. Even though ~5–10% outliers in the automatic contours need manual correction at visual checking, automatic contour propagation saves time to calculate OAR motion and the IRV. Since the outliers often occur at the superior or inferior of an OAR, only the portion of an OAR near the mobile tumor needs a careful inspection to incorporate intrafraction motion into the PRV by the tumor.

The T2W 4DMRI provides high‐soft tissue contrast with many fine anatomic landmarks in the image, facilitating both manual and automatic OAR contour quality. Moreover, using the internal navigator for 4DMRI image reconstruction, binning artifacts are almost negligible.^23^ Therefore, the accuracy and reliability of the DIR‐based propagated contours among 4DMRI images are expected to be higher (Fig. 2A) than 4DCT images with low soft‐tissue contrast and frequent binning artifacts.^28^, ^49^, ^50^ This explains the low intra‐observer (Fig. 1 and Table 1) and inter‐observer (Fig. 2B and Table 2) variability. In addition, the contour guideline is important, especially the superior boundary of the heart, defined as when the two ascending arteries split in axial view***.*** The uncertainty in heart contour also results from whether to include the fat layer inferior to the heart visible in T2W 4DMRI while unclear in 4DCT. The stomach contours have the highest uncertainty likely due to the interference from foreign objects and trapped air in the hollow structure. Studies of OAR segmentation were mostly based on CT or 4DCT.^51^, ^52^

### 4.2 Consistency and reliability of automatic OAR volumes using DIR mapping

The uncertainty of the free‐form DIR algorithm was reported to be ~3.5 mm in 4DCT in the thoracic and upper abdomen.^46^ Given more visible landmarks and fewer artifacts in T2W 4DMRI image,^23^ it is expected that the DIR uncertainty should be reduced. However, the image resolution of 4DMRI (2 × 2 × 5 mm^3^) is inferior to 4DCT (1 × 1 × 3 mm^3^), therefore, the DIR accuracy may be affected, so does the automatically propagated contours. On the other hand, the binning artifacts in 4DMRI are much fewer than 4DCT (see more discussion below). Overall, the quality of DIR alignment in 4DMRI should be similar to that in 4DCT,^46^ if not better, as shown in Fig. 2A. In addition, the major bronchial tree structure is seen in the thorax, facilitating lung DIR alignment. The liver contour has few artifacts because it is near the navigator (Fig. 2A). Mild binning artifacts are observed in the heart and stomach due to cardiac and digestive motions,^23^ which do not synchronize with respiration. The observed large IRV increase for the stomach (48 ± 20%) comparing to that of the liver (24 ± 9%) may result from highly heterogeneity inside the stomach but less clear organ boundary, possible internal gas compression and movement, and therefore higher uncertainties in the organ delineation.

As volunteer subjects are the surrogates of patients, in the presence of tumor or metastasis the OAR appearance may vary, including the tumor and possible edema. These extra objects, in fact, could be used as additional anatomic landmarks to facilitate the DIR and automatic contour propagation. Therefore, we expect that the results of healthy subjects can be applied to patients.

### 4.3 Contour variation resulted from 4DMRI binning artifacts

OAR contour similarity between two 4DMRI images that were reconstructed using the same image dataset but two concurrent respiratory surrogates (internal navigator and external bellows) is evaluated. Binning artifacts appear fewer in navigator‐triggered than bellows‐rebinned 4DMRI images,^23^ thereby enhancing the contour quality (*p* < 0.05, Fig. 4). The superior contour quality implies that the contour quality in navigator‐triggered 4DMRI is better than that of 4DCT where an external surrogate is almost always used. In fact, binning artifacts are commonly present in 4DCT^49^, ^50^ and can cause up to 90–110% gross tumor volume changes in lung cancer.^53^, ^54^ The high 4DMRI image quality and high soft‐tissue contrast improve that DIR and therefore contribute to the high OAR contour quality.

The automatically propagated contour quality from this MRI study is among the high ends with only 5–10% manual correction in comparison with 20–45% in 4DCT,^55^ which uses an external surrogate for reconstruction. Although the percentage is drastically reduced, a further investigation should be conducted to facilitate clinical applications in assessing OAR motion for IRV determination. As the contour outliers often occur in slices with drastic shape changes (large motions), such as the inferior lungs, they are likely caused by contour interpolation from stretched voxels to a slice. In addition, a different DIR algorithm may also be evaluated.

### 4.4 The OAR voxel probability within the IRV

The current ITV planning approach does not account for the OAR motion and therefore the planned dose to the OAR may not be accurate. Clinically, the only inter‐fractional variability in the OAR position relative to the tumor is considered using the PRV. In SBRT, OAR toxicity is often the limiting factor, preventing the prescription of a potent ablative dose with accelerated fractionation.^4^, ^5^ Therefore, accurate estimation of OAR dose is essential to optimize the SBRT dose prescription and to establish the OAR dose‐toxicity relationship for an improved therapeutic ratio.^13^

The introduction of IRV into SBRT planning is one way to account for OAR motion and to assess the OAR dose more accurately. Using voxel probability of OAR in or near the radiation field is another method because the OAR dose depends on the duration of the OAR move into or near the planning tumor volume (ITV+margin). Further investigation is needed to reduce the outliers in automatic organ delineation, to provide more accurate dose evaluation to the OARs, and to account for breathing irregularities that affect the ITV and IRV delineation using the time‐resolved 4DMRI technique over multi‐breathing cycles.^26^

## 5 CONCLUSION

Using T2W navigator‐triggered RC‐4DMRI and free‐form DIR algorithm, the automatic contours of four common OARs are evaluated with the STAPLE analysis and found to be indistinguishable with the manual contours for the lungs, heart, liver, and stomach in 10 subjects. High average contour similarity (0.89), sensitivity (0.92), and specificity (0.97) are observed. The external‐bellows‐rebinned 4DMRI provides 4% lower similarity on average, due to motion irregularity‐induced binning artifacts. Further investigations to reduce the manual correction rate (5–10%) and to evaluate the dosimetry consequences of volume increase from the OAR to IRV (20–50%) are needed prior to clinical applications.

## CONFLICT OF INTEREST

MSKCC has a Master Research Agreement with Philips Healthcare in clinical MR research for radiotherapy.

## References

[1] Keall PJ , Mageras GS , Balter JM , et al. The management of respiratory motion in radiation oncology report of AAPM Task Group 76. Med Phys. 2006;33:3874–3900. 17089851 10.1118/1.2349696

[2] Li G , Citrin D , Camphausen K , et al. Advances in 4D medical imaging and 4D radiation therapy. Technol Cancer Res Treat. 2008;7:67–81. 18198927 10.1177/153303460800700109

[3] Li G , Mageras G , Dong L , Mohan R . Image‐guided radiation therapy In: Khan FM, Gerbi BJ, eds. Treatment Planning in Radiation Oncology. Philadelphia, PA: Lippincott Williams & Wilkins; 2016:229–258.

[4] Timmerman R , McGarry R , Yiannoutsos C , et al. Excessive toxicity when treating central tumors in a phase II study of stereotactic body radiation therapy for medically inoperable early‐stage lung cancer. J Clin Oncol. 2006;24:4833–4839. 17050868 10.1200/JCO.2006.07.5937

[5] Timmerman RD , Herman J , Cho LC . Emergence of stereotactic body radiation therapy and its impact on current and future clinical practice. J Clin Oncol. 2014;32:2847–2854. 25113761 10.1200/JCO.2014.55.4675 PMC4152712

[6] Underberg RW , Lagerwaard FJ , Slotman BJ , Cuijpers JP , Senan S . Use of maximum intensity projections (MIP) for target volume generation in 4DCT scans for lung cancer. Int J Radiat Oncol Biol Phys. 2005;63:253–260. 16111596 10.1016/j.ijrobp.2005.05.045

[7] Adamson J , Chang Z , Wang Z , Yin FF , Cai J . Maximum intensity projection (MIP) imaging using slice‐stacking MRI. Med Phys. 2010;37:5914–5920. 21158304 10.1118/1.3503850

[8] Callahan J , Kron T , Schneider‐Kolsky M , et al. Validation of a 4D‐PET maximum intensity projection for delineation of an internal target volume. Int J Radiat Oncol Biol Phys. 2013;86:749–754. 23601897 10.1016/j.ijrobp.2013.02.030

[9] McKenzie A , van Herk M , Mijnheer B . Margins for geometric uncertainty around organs at risk in radiotherapy. Radiother Oncol. 2002;62:299–307. 12175561 10.1016/s0167-8140(02)00015-4

[10] Panakis N , McNair HA , Christian JA , et al. Defining the margins in the radical radiotherapy of non‐small cell lung cancer (NSCLC) with active breathing control (ABC) and the effect on physical lung parameters. Radiother Oncol. 2008;87:65–73. 18267345 10.1016/j.radonc.2007.12.012

[11] Topolnjak R , Borst GR , Nijkamp J , Sonke JJ . Image‐guided radiotherapy for left‐sided breast cancer patients: geometrical uncertainty of the heart. Int J Radiat Oncol Biol Phys. 2012;82:e647–655. 22270162 10.1016/j.ijrobp.2011.08.024

[12] Crane C.H . Hypofractionated ablative radiotherapy for locally advanced pancreatic cancer. J Radiat Res 2016;57:i53–i57. 27029741 10.1093/jrr/rrw016 PMC4990109

[13] Martin S , Johnson C , Brophy M , et al. Impact of target volume segmentation accuracy and variability on treatment planning for 4D‐CT‐based non‐small cell lung cancer radiotherapy. Acta Oncol. 2015;54:322–332. 25350526 10.3109/0284186X.2014.970666

[14] Timmerman R , Bastasch M , Saha D , Abdulrahman R , Hittson W , Story M . Optimizing dose and fractionation for stereotactic body radiation therapy. Normal tissue and tumor control effects with large dose per fraction. Front Radiat Ther Oncol. 2007;40:352–365. 17641519 10.1159/000106046

[15] Timmerman RD , Park C , Kavanagh BD . The North American experience with stereotactic body radiation therapy in non‐small cell lung cancer. J Thorac Oncol. 2007;2:S101–112. 17603304 10.1097/JTO.0b013e318074e4fa

[16] Milano MT , Constine LS , Okunieff P . Normal tissue toxicity after small field hypofractionated stereotactic body radiation. Radiat Oncol. 2008;3:36. 18976463 10.1186/1748-717X-3-36 PMC2596155

[17] Dawood O , Mahadevan A , Goodman KA . Stereotactic body radiation therapy for liver metastases. Eur J Cancer. 2009;45:2947–2959. 19773153 10.1016/j.ejca.2009.08.011

[18] King CR , Brooks JD , Gill H , Presti JC Jr . Long‐term outcomes from a prospective trial of stereotactic body radiotherapy for low‐risk prostate cancer. Int J Radiat Oncol Biol Phys. 2012;82:877–882. 21300474 10.1016/j.ijrobp.2010.11.054

[19] Cai J , Chang Z , Wang Z , Paul Segars W , Yin FF . Four‐dimensional magnetic resonance imaging (4D‐MRI) using image‐based respiratory surrogate: a feasibility study. Med Phys. 2011;38:6384–6394. 22149822 10.1118/1.3658737 PMC4108683

[20] Hu Y , Caruthers SD , Low DA , Parikh PJ , Mutic S . Respiratory amplitude guided 4‐dimensional magnetic resonance imaging. Int J Radiat Oncol Biol Phys. 2013;86:198–204. 23414769 10.1016/j.ijrobp.2012.12.014 PMC3628273

[21] Du D , Caruthers SD , Glide‐Hurst C , et al. High‐quality t2‐weighted 4‐dimensional magnetic resonance imaging for radiation therapy applications. Int J Radiat Oncol Biol Phys. 2015;92:430–437. 25838186 10.1016/j.ijrobp.2015.01.035 PMC4431950

[22] Glide‐Hurst CK , Kim JP , To D , et al. Four dimensional magnetic resonance imaging optimization and implementation for magnetic resonance imaging simulation. Pract Radiat Oncol. 2015;5:433–442. 26419444 10.1016/j.prro.2015.06.006

[23] Li G , Wei J , Olek D , et al. Direct comparison of respiration‐correlated four‐dimensional magnetic resonance imaging reconstructed using concurrent internal navigator and external bellows. Int J Radiat Oncol Biol Phys. 2017;97:596–605. 28011048 10.1016/j.ijrobp.2016.11.004 PMC5288126

[24] Harris W , Ren L , Cai J , Zhang Y , Chang Z , Yin FF . A technique for generating volumetric cine‐magnetic resonance imaging. Int J Radiat Oncol Biol Phys. 2016;95:844–853. 27131085 10.1016/j.ijrobp.2016.02.011 PMC4852391

[25] Stemkens B , Tijssen RH , de Senneville BD , Lagendijk JJ , van den Berg CA . Image‐driven, model‐based 3D abdominal motion estimation for MR‐guided radiotherapy. Phys Med Biol. 2016;61:5335–5355. 27362636 10.1088/0031-9155/61/14/5335

[26] Li G , Wei J , Kadbi M , et al. Novel super‐resolution approach to time‐resolved volumetric 4‐dimensional magnetic resonance imaging with high spatiotemporal resolution for multi‐breathing cycle motion assessment. Int J Radiat Oncol Biol Phys. 2017;98:454–462. 28463165 10.1016/j.ijrobp.2017.02.016 PMC5481849

[27] Yang YH , Zhou SJ , Shang P , Qi E , Wu SB , Xie YQ . Contour propagation using feature‐based deformable registration for lung cancer. Biomed Res Int. 2013;2013:701514. 24364036 10.1155/2013/701514 PMC3865642

[28] Murphy MJ , Wei Z , Fatyga M , et al. How does CT image noise affect 3D deformable image registration for image‐guided radiotherapy planning? Med Phys. 2008;35:1145–1153. 18404949 10.1118/1.2837292

[29] Lin L , Shi C , Liu Y , Swanson G , Papanikolaou N . Development of a novel post‐processing treatment planning platform for 4D radiotherapy. Technol Cancer Res Treat. 2008;7:125–132. 18345701 10.1177/153303460800700205 PMC2664298

[30] Faggiano E , Fiorino C , Scalco E , et al. An automatic contour propagation method to follow parotid gland deformation during head‐and‐neck cancer tomotherapy. Phys Med Biol. 2011;56:775–791. 21239848 10.1088/0031-9155/56/3/015

[31] Thornqvist S , Petersen JB , Hoyer M , Bentzen LN , Muren LP . Propagation of target and organ at risk contours in radiotherapy of prostate cancer using deformable image registration. Acta Oncol. 2010;49:1023–1032. 20831491 10.3109/0284186X.2010.503662

[32] Zambrano V , Furtado H , Fabri D , et al. Performance validation of deformable image registration in the pelvic region. J Radiat Res. 2013;54(Suppl 1):i120–128. 23824115 10.1093/jrr/rrt045 PMC3700513

[33] Guckenberger M , Sweeney RA , Wilbert J , et al. Image‐guided radiotherapy for liver cancer using respiratory‐correlated computed tomography and cone‐beam computed tomography. Int J Radiat Oncol Biol Phys. 2008;71:297–304. 18406894 10.1016/j.ijrobp.2008.01.005

[34] Li H , Chen HC , Dolly S , et al. An integrated model‐driven method for in‐treatment upper airway motion tracking using cine MRI in head and neck radiation therapy. Med Phys. 2016;43:4700. 27487887 10.1118/1.4955118

[35] He B , Huang C , Sharp G , et al. Fast automatic 3D liver segmentation based on a three‐level AdaBoost‐guided active shape model. Med Phys. 2016;43:2421. 27147353 10.1118/1.4946817

[36] van der Put RW , Kerkhof EM , Raaymakers BW , Jurgenliemk‐Schulz IM , Lagendijk JJ . Contour propagation in MRI‐guided radiotherapy treatment of cervical cancer: the accuracy of rigid, non‐rigid and semi‐automatic registrations. Phys Med Biol. 2009;54:7135–7150. 19904036 10.1088/0031-9155/54/23/007

[37] Feng Y , Kawrakow I , Olsen J , et al. A comparative study of automatic image segmentation algorithms for target tracking in MR‐IGRT. J Appl Clin Med Phys. 2016;17:5820. 10.1120/jacmp.v17i2.5820 PMC5875567 27074465

[38] Gou S , Lee P , Hu P , Rwigema JC , Sheng K . Feasibility of automated 3‐dimensional magnetic resonance imaging pancreas segmentation. Adv Radiat Oncol. 2016;1:182–193. 27868105 10.1016/j.adro.2016.05.002 PMC5113135

[39] Henke L , Kashani R , Yang D , et al. Simulated online adaptive magnetic resonance‐guided stereotactic body radiation therapy for the treatment of oligometastatic disease of the abdomen and central thorax: characterization of potential advantages. Int J Radiat Oncol Biol Phys. 2016;96:1078–1086. 27742541 10.1016/j.ijrobp.2016.08.036 PMC5376349

[40] Warfield SK , Zou KH , Wells WM . Simultaneous truth and performance level estimation (STAPLE): an algorithm for the validation of image segmentation. IEEE Trans Med Imaging. 2004;23:903–921. 15250643 10.1109/TMI.2004.828354 PMC1283110

[41] Peroni M , Spadea MF , Riboldi M , et al. Validation of automatic contour propagation for 4D treatment planning using multiple metrics. Technol Cancer Res Treat. 2013;12:501–510. 23745788 10.7785/tcrt.2012.500347

[42] Hong TS , Bosch WR , Krishnan S , et al. Interobserver variability in target definition for hepatocellular carcinoma with and without portal vein thrombus: radiation therapy oncology group consensus guidelines. Int J Radiat Oncol Biol Phys. 2014;89:804–813. 24969794 10.1016/j.ijrobp.2014.03.041 PMC4285340

[43] Martin S , Brophy M , Palma D , et al. A proposed framework for consensus‐based lung tumour volume auto‐segmentation in 4D computed tomography imaging. Phys Med Biol. 2015;60:1497–1518. 25611494 10.1088/0031-9155/60/4/1497

[44] Choi W , Xue M , Lane BF , et al. Individually optimized contrast‐enhanced 4D‐CT for radiotherapy simulation in pancreatic ductal adenocarcinoma. Med Phys. 2016;43:5659–5666. 27782710 10.1118/1.4963213 PMC5035305

[45] Zhang Q , Pevsner A , Hertanto A , et al. A patient‐specific respiratory model of anatomical motion for radiation treatment planning. Med Phys. 2007;34:4772–4781. 18196805 10.1118/1.2804576

[46] Liu F , Hu Y , Zhang Q , Kincaid R , Goodman KA , Mageras GS . Evaluation of deformable image registration and a motion model in CT images with limited features. Phys Med Biol. 2012;57:2539–2554. 22491010 10.1088/0031-9155/57/9/2539 PMC3349335

[47] Yang J , Woodward WA , Reed VK , et al. Statistical modeling approach to quantitative analysis of interobserver variability in breast contouring. Int J Radiat Oncol Biol Phys. 2014;89:214–221. 24613812 10.1016/j.ijrobp.2014.01.010 PMC3997068

[48] Li G , Arora NC , Xie H , et al. Quantitative prediction of respiratory tidal volume based on the external torso volume change: a potential volumetric surrogate. Phys Med Biol. 2009;54:1963–1978. 19265201 10.1088/0031-9155/54/7/007 PMC7356673

[49] Yamamoto T , Langner U , Loo BW Jr , Shen J , Keall PJ . Retrospective analysis of artifacts in four‐dimensional CT images of 50 abdominal and thoracic radiotherapy patients. Int J Radiat Oncol Biol Phys. 2008;72:1250–1258. 18823717 10.1016/j.ijrobp.2008.06.1937 PMC2583232

[50] Li G , Caraveo M , Wei J , et al. Rapid estimation of 4DCT motion‐artifact severity based on 1D breathing‐surrogate periodicity. Med Phys. 2014;41:111717. 25370631 10.1118/1.4898602 PMC4241828

[51] Schreibmann E , Marcus DM , Fox T . Multiatlas segmentation of thoracic and abdominal anatomy with level set‐based local search. J Appl Clin Med Phys. 2014;15:22–38. 10.1120/jacmp.v15i4.4468 PMC5875508 25207393

[52] Thomson D , Boylan C , Liptrot T , et al. Evaluation of an automatic segmentation algorithm for definition of head and neck organs at risk. Radiat Oncol. 2014;9:173. 25086641 10.1186/1748-717X-9-173 PMC4123306

[53] Persson GF , Nygaard DE , Brink C , et al. Deviations in delineated GTV caused by artefacts in 4DCT. Radiother Oncol. 2010;96:61–66. 20570002 10.1016/j.radonc.2010.04.019

[54] Li G , Cohen P , Xie H , Low D , Li D , Rimner A . A novel four‐dimensional radiotherapy planning strategy from a tumor‐tracking beam's eye view. Phys Med Biol. 2012;57:7579–7598. 23103415 10.1088/0031-9155/57/22/7579

[55] Thor M , Petersen JB , Bentzen L , Hoyer M , Muren LP . Deformable image registration for contour propagation from CT to cone‐beam CT scans in radiotherapy of prostate cancer. Acta Oncol. 2011;50:918–925. 21767192 10.3109/0284186X.2011.577806

